# A V530I Mutation in c-KIT Exon 10 Is Associated to Imatinib Response in Extraabdominal Aggressive Fibromatosis

**DOI:** 10.1155/2010/458156

**Published:** 2010-03-17

**Authors:** Jean-Emmanuel Kurtz, Irène Asmane, Anne-Claire Voegeli, Agnès Neuville, Armelle Dufresne, Valère Litique, Christine Chevreau, Jean-Pierre Bergerat

**Affiliations:** ^1^Pôle d'Hématologie et d'Oncologie, Hôpitaux Universitaires de Strasbourg, 67098 Strasbourg, France; ^2^Pôle de Biologie, Hôpitaux Universitaires de Strasbourg, 67098 Strasbourg, France; ^3^Service d'Anatomie pathologique, Hôpitaux Universitaires de Strasbourg, 67098 Strasbourg, France; ^4^Service d'Oncologie Médicale, Centre Hospitalier de Chambéry, 73011, France; ^5^Service d'Oncologie Médicale, Centre Hospitalier Universitaire, 31052 Toulouse, France

## Abstract

Aggressive fibromatosis (AF) or desmoid tumor is a rare condition, characterized by deep tissue invasion by a monoclonal fibroblastic neoplasm, developed from musculoaponeurotic structures. Surgery is the treatment of choice, but negative margins can hardly been achieved in large tumors, and can lead to major functional disability. AF medical therapy includes nonsteroids anti-inflammatory drugs, tamoxifen, with inconsistent results. Several reports of imatinib efficacy in AF appear in the literature. Here, we describe for the first time a V530I *KIT* exon 10 mutant that was associated to a dramatic imatinib response in an extraabdominal aggressive fibromatosis. The previously discovered V530I substitution was characterized in the core binding factor AML, but had never been reported in any other condition, so far. In this paper, we discuss the *KIT* exon 10 mutations or polymorphisms that have been described in a variety of KIT-related conditions, including acute myelogenous leukemia, mastocytosis, and aggressive fibromatosis.

## 1. Case Report

**Figure 1:**
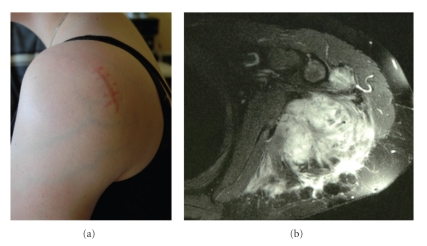
Clinical findings at diagnosis. Note the bulky mass of the shoulder with collateral circulation (a). MRI (T1 gadolinium sequences) shows a heterogeneous tumor infiltrating muscles and subcutaneous tissue (b).

A 22-year-old Caucasian female without any particular medical history suffered from a traumatic fracture of the left humerus great tuberosity in 2002. She underwent a non-operative treatment with a Mayo-Clinic splint and was discharged from the hospital. Radiologic outcome wasunremarkable, though she complained with persistent shoulder pain. Three years later, a painful mass of the shoulder rapidly appeared, impairing shoulder mobility. She was referred to our institution. Clinical examination revealed a hard, bulky mass of the shoulder, with collateral circulation. The tumor involved the whole curving contour of the shoulder, and reached the axilla at its posterior limit. Mobilization of the arm was almost impossible due to both pain and stiffness (Figure 1(a)).A shoulder MRI revealed a 12 × 6 cm irregular, heterogeneous mass of the posterior part of the shoulder (Figure 1(b)). The tumor invaded subcutaneous tissue as well as the deltoid and infrasupinatous muscles. Necrotic areas diagnosed as T1-hyposignal were found, as well as other tumor areas that were strongly enhanced by contrast injection. There was no apparent bone destruction. A surgical biopsy was performed, diagnosing aggressive fibromatosis (extraabdominal desmoids tumor).

**Figure 2:**
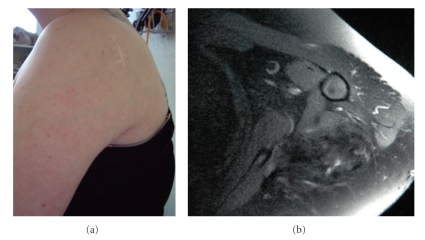
Clinical response after Imatinib therapy: Recovery of the whole curving contour of the shoulder, with decrease of collateral circulation (a). MRI findings: T1 gadolinium sequences confirming the tumor very good partial response with disappearance of tumor-related hypersignal (b).

Due to the tumor burden, curative surgery could not be performed, and a medical therapy consisting in imatinib mesylate (Glivec) was started at a daily dose of 400 mg. After 4 weeks of treatment, the patient noticed an improvement in the abduction of the arm as well as a slight tumor size decrease. The treatment was continued at the same dosing, with consistent improvement in mobility, tumor measurements and analgesics consumption. There was no significant side-effects. The treatment was finally stopped at month 34, in the setting of tumor regression, complete recovery of arm mobility and function and complete discontinuation of analgesics (Figure 2(a)). Control MRI of the shoulder confirmed the very good partial response (Figure 2(b)). At a follow-up of 42 months (1 year off-therapy), the patient remains tumor and symptoms-free, without any tumor re-growth.

**Figure 3:**
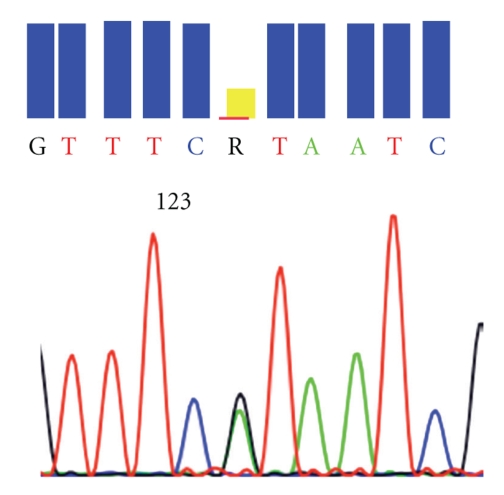
*KIT* exon 10 point mutation 1609 G→A (resulting in the amino-acid substitution V530I).

In the light of such a dramatic response to imatinib, c-kit exon 10 was fully sequenced from the tumor frozen samples. 50 ng of genomic DNA was amplified by PCR in 50 *μ*l reaction volume containing 2,5 U AmpliTaq Gold DNA polymerase (Applied Biosystems, Forster city, CA), 0,2 mM dNTP, 1,5 mM MgCl_2_, and 0,2 *μ*m of the forward and reverse primers 5′-ATCCCATCCTGCCAAAGTT-3′ and 5′-CTGTGGGGAGAAAGGGAAA-3′, respectively, flanking exon 10. PCR products were verified by electrophoresis, showing a 246 bp amplified fragment, purified by using Microcon-PCR Filter Unit (Millipore, Paris, France) and directly sequenced with the Big Dye Terminator v1.1 Cycle sequencing kit (Applied Biosystems, Forster city, CA). finally the PCR product was analyzed on ABI PRISM 3100 Genetic Analyser (Applied Biosystems, Forster city, CA). The sequences were aligned with the GB sequence of human CKIT (locus HSU63834) using the software Seqscape v2.5 (Applied Biosystems, Forster city, CA). All sequencing reactions were performed in both forward and reverse directions, and the mutation was confirmed by a second sequencing on an independent PCR, revealing a V530I mutation in the transmembrane domain of c-kit (Figure 3).

## 2. Discussion

Aggressive fibromatosis (AF) is a monoclonal disease, arising from deep musculoaponeurotic structures [1]. In spite of being nonmalignant, the outcome of AF is characterized by local aggressiveness, making curative surgery a difficult challenge, as patients are exposed to local recurrence. The clinical outcome of AF is unpredictable, and many treatment options have been considered, including surgery, radiation therapy and medical therapy. Systemic medical therapy has focused on NSAIDs such as Sulindac and anti-Cox2 as well as hormonal therapy (tamoxifen) although AF inconsistently express estrogen receptors [2]. 

The KIT gene encodes the stem cell growth factor receptor, a type III transmembrane receptor tyrosine kinase that has been involved in the pathogenesis of various conditions, including gastro-intestinal stromal tumors (GISTs), mastocytosis, acute myelogenous leukemia (AML) as well as piebaldism, a rare autosomal inherited skin disorder. The tyrosine kinase inhibitor imatinib mesylate (Glivec) has become a cornerstone of advanced GIST, in which KIT exon 11 mutations are associated with imatinib efficacy, as opposed to these that are present in exon 9 [3]. However, *KIT* exon 10 mutations have not been reported so far in GISTs, as opposed to deletions at the intron10-exon11 boundary that may impact response to imatinib [4].

Imatinib has been incorporated in the therapeutic armamentarium of AF, in the light of several case reports of long-lasting responses [5–8]. However, all AF do not respond to imatinib treatment, as shown in a retrospective analysis of 19 cases of heavily pretreated patients in whom the response rate was only 15.7% (3/19 PR) [8]. Interestingly, it is unlikely that positive *KIT* staining would predict imatinib sensitivity as in this series, most of the cases were *KIT* negative. Molecular determinants of tumor response to tyrosine kinase inhibitors are well known in gastro-intestinal stromal tumors, where *KIT* mutations are predictive for imatinib efficacy [3]. In the retrospective series reported by Heinrich et al., no *KIT* mutation was however found, even in the three responders to imatinib [8]. This data contrasts with previous reports of *KIT* exon 10 point mutation 1621 A→C (resulting in the amino-acid substitution M541L) that could predict imatinib sensitivity [6]. Similar findings were reported in a French Sarcoma Group study, where 3 out of 10 patients experiencing tumor control with imatinib actually had an M541L mutation [9]. Moreover, Seinfeld et al. reported the occurrence of a limited clinical response, again in a M541L patient [10]. However, contradictory data also appear in the literature. In the light of experiments conducted by Tamborini et al., as well as Bertucci et al., it was hypothesized that the exon 10 M541L variant is not activating neither inactivating and is actually a *KIT* polymorphism [11, 12]. Consistent findings were reported by Krüger et al., who found that the allele frequency of the KIT variant M541L was as high as 8.1% in the healthy population, not differing from chronic myelogenous patients (8.3%) [13]. The V530I variant has to our knowledge, never been described so far, in either AF or other KIT-related diseases such as mastocytosis, GISTs or piebaldism, [14, 15]. The exon 10 V530I substitution alters the transmembrane domain of *KIT* (contained between amino acids 521–543), and was described in acute myelogenous leukemia [16, 17]. Similar and rare (<5%) exon 10 KIT-activating transmembrane mutations such as A533D or F522C have been reported in mastocytosis [15]. *KIT* deletions or insertions appear in 7 to 17% of AML cases (raising to 33% in the setting of chromosome 16 inversion), affecting members of the core binding factor (CBF) gene family [16–18]. In a series of 103 AML patients, those harboring a KIT mutation had a shorter event-free and relapse-free survival [18]. There is little data with regard to imatinib therapy in AML patients with CBF *KIT* mutations. In a series of 3 patients, imatinib had transient activity in a patient with exon 8 in-frame deletion, but none in the 2 patients carrying D816Y and D816V mutations [19]. In a series of published by Cortes et al., 18 patients with recurrent or refractory AML or myelodysplastic syndrome were challenged with imatinib, with very transient results [20]. Unfortunately, no KIT analysis was performed in this study. A trial investigating dasatinib, another tyrosine kinase inhibitor is currently recruiting AML patients harboring CBF mutations in France. Interestingly, V530I variants were associated, in this condition with a greater imatinib sensitivity, as the IC_50_ was lower than the wild-type *KIT* by a 5-fold factor. Moreover, inhibition of V530I variants resulted in both proliferation abrogation and increased apoptosis in vitro [16]. We have not investigated whether our patient exhibited a germline or a somatic mutation as tissue sampling consisted in tumor biopsy only, and no consent was obtained to test for any germline mutation in our patient who lacked significant familial history of cancer. There is, moreover, no data in the literature supporting the hypothesis that the V530I KIT (as opposed to the aforementioned M541L) mutation could be a polymorphism rather than a rare, highly penetrating alteration. 

Our patient exhibited a typical medical history of trauma, preceding the onset of AF by an interval of three years, a delay consistent with literature data [2]. There is little data in the literature regarding imatinib treatment duration in AF. Of note, in our case, evidence for treatment efficacy was present as soon as 4 weeks after treatment started. Moreover, imatinib was administered for 3 years as the tumor size constantly decreased during this period. However, and as opposed to the report by Wcislo et al., imatinib was then stopped without subsequent local relapse [7]. In other reports, the duration of imatinib therapy varied between 34 weeks and 16 months [5, 6, 10].

Based on the literature data, as well as this report, it appears mandatory that AF patients be screened not only for M541L substitution, which physiopathology remains at the least, controversial, but should undergo systematic exon 10 sequencing to detect transmembrane variants, such as V530I. Hence, the presence of such a mutation could support front-line imatinib therapy, rather than more classic strategies, including tamoxifen or NSAIDs. Dramatic response to imatinib is clearly associated with this substitution, and provided such a mutation has been detected, not only imatinib can be considered, but also other TK inhibitors, such as sunitinib or others [21]. Whether the V530I variant might successfully be challenged with tyrosine kinase inhibitors other than imatinib is unknown. Growney et al. tried to assess the efficacy of PKC412, a novel tyrosine kinase inhibitor in a variety of *KIT* mutants, including V530I, but this variant could not transform the Ba/F3 cells in their experiment [21]. Interestingly, reports of sunitinib efficacy in imatinib-resistant AF patients appear in the literature, suggesting that as in GISTs, treatment strategies with “rescue” alternate TK inhibitors can be considered in selected patients [22].
